# The Impact of Sensory, Motor and Pain Impairments on Patient- Reported and Performance Based Function in Carpal Tunnel Syndrome

**DOI:** 10.2174/1874325001711011258

**Published:** 2017-11-10

**Authors:** Goris Nazari, Niyati Shah, Joy C MacDermid, Linda Woodhouse

**Affiliations:** 1 Physiotherapy, Health & Rehabilitation Science, London, Ontario, Canada; 2 School of Rehabilitation Science, McMaster University, Hamilton, Ontario, Canada; 3 Physical Therapy, University of Alberta, Edmonton, Alberta, Canada

**Keywords:** Carpal Tunnel Syndrome, Function, Dexterity, Quick DASH, Sensorimotor measures

## Abstract

**Background:**

Research has suggested that persistent sensory and motor impairments predominate the symptoms experienced by patients with carpal tunnel syndrome (CTS); with intermittent pain symptoms, being less predominant.

**Objective:**

The study aims to determine the relative contribution of sensory, motor and pain impairments as contributors to patient-report or performance-based hand function.

**Methods:**

Fifty participants with a diagnosis of CTS confirmed by a hand surgeon and electrodiagnosis were evaluated on a single occasion. Impairments were measured for sensibility, pain and motor performance. A staged regression analysis was performed. In the first step, variables with each of the 3 impairment categories were regressed on the Symptom Severity Scale (SSS) to identify the key variables from this domain. Models were created for both self report (Quick Disabilities of arm, shoulder and hand- Quick DASH) and performance based (Dexterity) functional outcomes. Backward regression modelling was performed for SSS and then, to allow comparability of the importance of different impairments across models, the 7 significant variables from the SSS model were forced into the models.

**Results:**

Variables: age, touch threshold and vibration threshold of the little finger of unaffected hand, median-ulnar vibration threshold ratio of affected hand, mean pain tolerance of unaffected hand, grip strength and pinch strength of affected hand, explained 31%, 36% and 63% of the variance in SSS, Quick DASH and dexterity scores, respectively.

**Conclusion:**

Hand function in patients with CTS is described by variables that reflect sensory status of the median and ulnar nerves, the persons pain threshold, grip and pinch strength impairments and age.

## 1 INTRODUCTION

Carpal Tunnel Syndrome (CTS) is an upper extremity neuropathy involving the compression of the median nerve at the area of the wrist, leading to symptoms like tingling, numbness, pain and weakness [1]. The population prevalence has been estimated at approximately 1.5-2% [2, 3]. Data produced by a Workplace Safety and Insurance Board, suggest that approximately 5% of all workplace injury claims were the result of CTS [4]. Both clinical [5, 6] and electrodiagnostic [7, 8] measures are commonly used, with a variety of diagnostic and assessment methods used [9]. Conservative and surgical treatments are both effective in use but the latter demonstrating a higher overall effectiveness and becoming the gold standard for the relief of symptoms upon failure of conservative management [10-13].

Since the pathology involves nerve compression, symptoms expected in CTS include pain, sensorimotor abnormalities and loss of hand function. Symptoms reported vary across clinical studies [1], but numbness, tingling and loss of strength are consistent findings in the CTS [9, 14]. In a descriptive qualitative study, patients with CTS reported that sensory symptoms were the most predominant aspect of their problem, bothersome and affected their function [15]. They reported difficulty in manipulation of small objects, lifting weights and performing other tasks of daily living. Patients with CTS also often complain of motor weakness [16]. Weak grip and pinch strength limit function and affect the overall quality of life [9]. In 1998, an attempt to arrive at a consensus definition of carpal tunnel syndrome for epidemiological studies suggested that “In the absence of electrodiagnostic findings, combinations of symptom characteristics and physical examination findings provide the greatest diagnostic information.’ This definition focused on the location of the symptoms rather than the nature of the symptoms allowing a wide spectrum of symptoms to be inclusive of the definition of CTS. Classic/probable CTS was defined as “Numbness, tingling, burning, or pain in at least 2 of digits 1, 2, or 3.” [17]. Fewer studies have focused on differentiating sensory paresthesia from pain. However, in a descriptive survey approximately half of patients with CTS reported not having any pain [8].

Based on our previous qualitative work, we identified three constructs that could contribute to hand function: sensory, motor and pain. The main objective of this study was to evaluate these constructs from a quantitative perspective and determine how sensory, pain and motor variables contribute to either performance-based or self-reported function.

## 2 Methods and Methods

### 2.1 Study Design

This was a cross-sectional study

### 2.2 Participants

Based on sample size calculation, fifty participants were required to conduct a multivariate analysis of up to 7 potential predictors. Participants were recruited from a list of patients waiting for surgical release of their carpal tunnel. All the participants had CTS confirmed by electrophysiology. Patients aged 18 years or older who had been diagnosed with CTS but had not yet undergone surgery were recruited. Understanding English was essential to understand some of the test procedures and hence was an inclusion criterion. All patients signed informed consent.

Of the 60 participants contacted, 50 met eligibility criteria and gave their consent to participation.

### 2.3 Independent Variables

The independent variables chosen for the study were divided into 3 categories based on the conceptual content they addressed:

#### 2.3.1 Sensory Variables

Sensory abnormalities were considered a predominant feature of CTS by patients. Two different QST measures were used to evaluate threshold for touch and vibration.

#### 2.3.2 Touch Threshold

Touch threshold has been used as a quantitative sensory measure test for patients with CTS [18]. Touch threshold was measured with the NK Pressure Specified Sensory Device (PSSD), a computerized hand-held device that allows the participant to signal when they feel a touch stimulus [1]. The PSSD has 65% specificity and 81% sensitivity in diagnosing CTS [19]. The grams of force required to perceive touch was recorded by a computer. The participant’s hand was laid on the tabletop, supported by moulded plastic, palmar surface up. The participant was then asked to close his or her eyes while the tester slowly lowered the PSSD, prongs perpendicular to the participant’s fingertips, onto the distal phalanx, applying enough pressure to slightly indent the skin. The participant pressed a trigger button held in the opposite hand as soon as the stimulus was perceived. The PSSD was applied five times with variation in site and timing. The lowest and highest values were dropped and the three remaining trials were averaged. The index (D2), and little (D5) fingers were tested on each hand to distinguish between ulnar and median nerve distribution. The units of measurement were grams per square millimetre (g/mm^2^).

#### 2.3.3 Vibration Threshold

Vibration threshold was evaluated using the J-Tech Vibrometer which measures the ability to feel a 50 Hertz stimulus [1]. The test protocol and the device that was used in this study have been evaluated for reliability in patients with CTS and have intra-class correlation coefficient (ICC) as high as 0.89 for test-retest [20]. The participant’s digit was placed lightly on the device’s vibrating pin. A sample stimulus was provided as practice before testing. During the test, a ramped protocol of intermittent vibration stimuli was applied to the digit. Participant indicated when the stimulus was perceived with a handheld trigger. The J-Tech software determines a threshold score after multiple cycles of forced choice responses. The index (D2) and little (D5) fingers were tested on each hand. The unit of measurement was micrometer. For data analysis, both raw and median-ulnar nerve ratio scores were entered. The median-ulnar nerve ratio was computed as the score for the index versus the little finger of the affected hand in order to determine the difference in threshold between median and ulnar nerve distribution pattern.

#### 2.3.4 Pain Variables

Pain is another common symptom reported by patient’s having CTS [1, 9]. Pain threshold and pain tolerance were tested for both hands. Pain algometry has been performed using a pressure device [21] with an ICC = 0.97 for test- retest reliability [22]. Pain threshold and tolerance for pressure stimulus were measured using the J-Tech algometer [1]. Participants were asked to lay their hand on the tabletop, palmar surface up. The tester applied the algometer onto the test site (located about the participant’s horizontal thumb width from the wrist crease) and the participant indicated when the pressure “became uncomfortable” (pain threshold). The tester continued to apply the algometer until the participant indicated that the pressure “became intolerable” (pain tolerance). The procedure was repeated 3 times to obtain an average value for pain threshold and pain tolerance. As for all of the measures, the procedure was conducted on both the hands in order to distinguish between the results of the affected and the unaffected hand. The unit of measurement was pounds per square cm (lbs/cm^2^).

#### 2.3.5 Motor Variables

Studies have shown that patients with CTS have impaired grip and pinch strength [1, 16]. Grip and pinch strength was assessed bilaterally. *Grip strength:* Grip strength was tested with NK digit grip device using the standardized positioning: the elbow flexed to 90 degrees, the forearm in neutral and the participants were asked to grip the NK digit grip device at the second handle position from the smallest grip size (American Society of Hand Therapists) [1].The participant was instructed to squeeze the device as hard as they could. Five trials were performed with a 10-second rest between each grip. Reliability is high for this test procedure with ICCs exceeding 0.87 for test-retest [23]. The lowest and highest values were dropped and the mean of the 3 remaining trials was noted. The procedure was then repeated on the opposite hand. The grip strength was measured in kilograms (0- 100 kg). *Pinch Strength:* Pinch strength testing was performed with the NK dynamometer-pinch device using the standardized positioning described by the American Society of Hand Therapists (ASHT): the elbow was flexed at 90 degrees, the forearm in neutral and the participant were asked to grip the NK pinch device in the key position [1]. These procedures have been shown to have high test-retest reliability with ICC’s> 0.90 [5]. The participant was instructed to squeeze the device as hard as they could. Five trials were performed with a 10-second rest between each pinch. The lowest and the highest values were dropped and the mean of the 3 remaining trials was recorded. The procedure was performed on both the affected and unaffected hands. Pinch strength was measured in kilograms (0-20 kg).

#### 2.3.6 Age

Age is considered to influence symptoms and function in patients with CTS [24]. Hence, age was included as an independent variable in the study. It was recorded as date of birth (yy/mm/dd).

### 2.4 Dependant Variables

Participants were assessed for their symptom severity, functional and dexterity abilities. The symptom severity scale was used as the dependent variable to reduce the number of impairments entered into the final regression models of function. Given that one functional measure was self-reported *(Quick DASH)* and one was performance-based (Dexterity), we elected to use a carpal tunnel specific symptom scale to reduce the pool of items; as a means of avoiding biasing the analysis towards one type of functional outcome measure versus the other.

#### 2.4.1 Symptom Severity Scale (SSS)

SSS is a part of the Boston Carpal Tunnel Questionnaire (BCTQ). This self report questionnaire was used in the study to understand the severity of the symptoms from the participant’s own perspective. The BCTQ was developed by Levine and colleagues in 1993 and has high reproducibility (Pearson correlation co-efficient r= 0.91) and internal consistency (cronbach alpha= 0.89) [25]. This questionnaire contains eleven questions with multiple choice responses, with a score ranging from 1 point (mildest) to 5 points (most severe). As an example, one of the questions on the scale is “Do you have tingling sensations in your hand?” and participants rate it on a scale of 1 to 5, where 1 is ‘no tingling’ and 5 is ‘very severe tingling’. The total symptom severity score was calculated as the mean of the scores for the eleven individual items [25]. The final score was out of 5.

#### 2.4.2 The Quick Disabilities of the Arm, Shoulder, Hand (Quick DASH)

Disability of the upper limb has been assessed for CTS patients using the validated DASH [26]. The majority of items on the DASH (21 out of 30) address functional tasks, five relate to symptoms and one item is dedicated to each of the four remaining concepts: social, work, sleep and capability. The DASH is presented as a uni-dimensional scale where a higher score indicates greater disability. A shorter version of the DASH, the 11-item Quick DASH, was used in this study [27]. Each item on the Quick DASH has a five-point response scale ranging from “no difficulty or no symptom” to “unable to perform activity or very severe symptom”. Like the DASH, Quick DASH also has 2 optional modules. The score was calculated as a mean of the main 11 items and then computed according to the formula provided. The Quick DASH scores cannot be calculated if there is more than one item with a missing score. The tool has high internal consistency (cronbach alpha ≥ 0.92) and high test-retest reliability (ICC ≥ 0.94) [26].

#### 2.4.3 Dexterity

Dexterity, or the ability to manipulate objects with the hand, is dependent on both sensory and motor function of the nerves. Problems manipulating small objects, is one of the most common complaints of patients with CTS [1, 9]. Thus, dexterity was chosen as a performance based functional outcome for the study. The NK dexterity test was used in the study. It is suitable for testing persons within the carpal tunnel population because it has separate subtests for small, medium and large objects [6, 28]. Small objects dexterity was tested in this study. The participant was seated at the board with the beginning of the small object set in line with the dominant hand. The objects included small threaded pins, small straight pins and washers and small pegged balls. The participant was instructed to complete a cycle of removing and replacing all the small objects in one shot, without a break. They were timed from the point they started to the point they completed one full cycle. Time was recorded in seconds. Each participant completed three timed trials and the mean time of the trials was calculated. The test was conducted bilaterally. Dexterity testing has fair to excellent intra-occasion reliability with ICC’s ranging from 0.53- 0.86 [28].

### 2.5 Analysis

Data was entered into statistical software PASW Statistics 18 (SPSS, Inc., 2009, Chicago, IL) and checked for quality by random rechecks of the database against the original data sheets. Linear regressions using Pearson correlations were used to look at bivariate relationships between variables. Multivariate backward regression was used to determine, if the chosen independent variables (sensory, motor, pain and age) were able to explain variance in each of the dependant variables (Symptom Severity, Quick DASH and hand dexterity). Regression analyses were conducted after testing the assumptions of normality, linearity and homoscedasticity. It was our intention to include at least one variable representing each of the three constructs (pain, motor or sensory) in our multivariate modeling of hand function. However, at the outset we had multiple variables that could be used to represent each of these constructs. Hence, the first data modeling use regression to reduce the number of pool of items within the construct to those most influential. There was multicollinearity in the pain, hence, only 1 pain variable “Pain tolerance mean of the unaffected hand” (p = 0.014, p ‹ 0.05) was chosen for entry into the modeling of function. After adjusting for multicollinearity, 7 factors were selected for regression modeling of function including age and variables representing sensory, motor and pain impairments. To allow us to compare the importance of different factors across the two functional constructs (self-reported versus performance) all seven variables were forced into the model. A table explaining the steps involved in analyses.

## 3 RESULTS

Subjects characteristics are presented in Table (**1**). In the regression modeling of Symptom Severity scale as a dependant variable, the sensory and motor variables did not show any multi- collinearity, however all the 4 pain variables demonstrated significant multi-collinearity. So, only 1 pain variable “Pain tolerance mean of the unaffected hand” was chosen as it was the most significant predictor amongst the 4 variables (p = 0.014, p ‹ 0.05). The sensory and motor variables were selected for the final model if they demonstrated a significant relationship to SSS at the conservative level of p<0.10. The 7 variables entered into the regression model were:

Age

Touch threshold of little finger of unaffected hand

Vibration Threshold of little finger of unaffected hand

Median Ulnar vibration threshold ratio of affected hand

Mean pain tolerance of unaffected hand

Grip strength of affected hand

Pinch strength of affected hand

Regression model including these 7 variables explained **31%** of the variance in Symptom Severity Score Table (**2**), **36%** variance in Quick DASH score Table (**3**) and **63%** variance in Dexterity score (Table **4**).

The total variance explained by all of the models was 63%. Only vibration threshold in the little finger of the unaffected hand and age where significant predictors of dexterity; with grip strength showing a trend towards. Vibration threshold of little finger of the affected hand was a significant correlate of both self-reported and function and hand dexterity; whereas age was also predictive of dexterity. (Tables **3** and **4**). Pain variables were not significant for either self-reported and function for dexterity.

## 4 DISCUSSION

The results of our study helped in determining the seven best variables that explain the variance in symptom severity and function. The 7 variables that went into the final regression model and explained the outcomes were age, touch threshold of little finger of unaffected hand, vibration threshold of little finger of unaffected hand, Median Ulnar vibration threshold ratio of affected hand, pain tolerance of unaffected hand, grip strength of affected hand and pinch strength of affected hand. These were the main findings of the study. To understand the impact of each of these variables and to see how it correlates with the existing literature, we would now discuss each of the variables in detail.

Age, as is well know influences function [24] and this explains the inclusion of this variable in the final model. Age was inversely related to function, i.e. with the increase in age the function decreased. This is in agreement with the existing literature, which indicates that with or without an existing disease condition, age influences function and increase in age relates to decreased functional capacity [24].

The next group was the sensory variables. The first two of the three sensory variables included in the model were touch threshold and vibration threshold, both measured from the little finger of the unaffected hand. As mentioned earlier, most of our participants (36/50) had bilateral symptoms. Thus, in our analysis we had labelled the more involved hand as the affected hand and the less involved as the unaffected. In our sample, for 23 participants the affected hand was not the dominant hand, which further explains the thresholds of unaffected hand being the influential variable. This correlates with findings from other studies which suggest that most activities of daily living require bilateral hand function [1, 7, 29] and thus even the unaffected hand influences the outcomes. Further, the inclusion of these two variables seemed counter-intuitive as they did not involve the median nerve (the influential variable was for little finger, which represents ulnar nerve distribution).We attribute this finding to the characteristics of our sample and the fact that ulnar and median nerve pathology often co-exists [30]. Moreover, since all of our patients were waiting for surgery, they had substantial median nerve compression. Thus, the extent of loss of sensibility in the median nerve may not have differentiated the functional level well in this sample. The touch threshold had a positive relation with SSS score and Quick DASH score. Higher touch threshold values co-related with higher scores on SSS and Quick DASH. Whereas, for the vibration threshold the relation was inverse and higher scores on the scales correlated with lower vibration threshold. These results are consistent with the literature findings for touch and vibration threshold measurements [18, 31] but provide additional information for CTS population as these have never been evaluated to determine their influence on function. The relations between dexterity, touch and vibration threshold were reversed when compared to that of SSS and Quick DASH. Dexterity demonstrated positive correlation with vibration threshold and negative correlation with touch threshold.

The median- ulnar vibration threshold ratio was the next variable and was computed to distinguish between the median and ulnar nerve distribution. Higher ratio value indicated higher vibration threshold for index finger (median nerve distribution) as compared to little finger (ulnar nerve distribution). This ratio variable proved to be significant for affected hand and was included in the model. The higher ratio suggested that vibration threshold was high for index finger of affected hand, implying marked sensory impairment [31]. Although this was contradictory to the initial findings of vibration threshold and no studies were found in the existing literature discussing such ratio variable, we decided to include it in the model as the correlation was more significant as compared to just vibration threshold.

Pain tolerance of the unaffected hand was the next variable in the final regression model. Our results suggest that the higher the pain tolerance the more severe are the symptoms (higher SSS final score), and the lower is the functional capacity (Quick DASH scores and dexterity). These results are in agreement with the literature that suggests pain thresholds correlate with function [21]. Our findings were however specifically related to CTS population as compared to the literature findings which were generalised to upper limb and torso.

The next 2 variables were grip and pinch strength of affected hand. These variables have been reported to influence function [22]. They are thus considered an important aspect of functional evaluation for patients with CTS and other upper extremity neuropathies. The results of our study suggested that grip strength had inverse relation with all 3 dependant variables, indicating that higher symptom severity scale and Quick DASH scores and higher timings on dexterity test (i.e. more difficulty with function) were related to reduced grip strength. For pinch strength however, the results suggested direct correlation with Quick DASH and inverse relations with SSS and dexterity scores. These pinch and grip strength scores and their influence on function indicate the importance of evaluating and managing motor symptoms in patients with CTS.

Overall, these 7 variables, explained **31%** variance in Symptom Severity Score Table (**2**), **36%** variance in Quick DASH score Table (**3**) and **63%** variance in Dexterity score (Table **4**).

### 4.1 Clinical Implications

The results of our study helped in identifying the best 7 variables that explain symptom severity and function in CTS patients. These variables explain significant variance (63%) in dexterity. However, for Symptom Severity Score and Quick DASH scores the same 7 variables are able to explain just over half that amount of variance (31% and 36% respectively). This limits the application of the results to clinical practice. Clinicians can consider measuring these 7 variables to understand the effect of CTS symptoms on dexterity, but can be less confident about their usefulness in understanding symptom severity or self reported function. Also, function was measured both as a performance based outcome as well as a self report outcome increasing the application of the results for clinicians using any type of functional outcomes. The study results partially fulfilled the purpose of the study as it did provide clinicians with a set of 7 variables to assess while considering dexterity as an outcome. Future longitudinal studies with the same variables are required to assess the utility of these measures in predicting dexterity, symptoms severity or function over time.

### 4.2 Limitations and Recommendations

Although the authors included variables with well established psychometric properties and used appropriate statistical analysis techniques, there are a few limitations to the study. The study sample included participants with CTS who were waiting for surgery, indicating that their symptoms were severe. This limits the clinical application of the results to that particular population. Moreover, the study was carried out as a cross sectional project and did not consider the impact of all the different measurements on change in functional status overtime. Also, the tests were performed in a single 3 hour long visit with functional outcomes measured towards the end. Again, this was based on participant priority and all the testing was carried out in a single visit to make it convenient for the participant. It is unclear if this had an impact on functional scores and would not be considered as a major limitation for the study. Also in the regression models for Quick DASH and dexterity the variables were forced in. A different approach allowing other variables in the model may give different results. Moreover, initially with the 19 variables in the model, the study was underpowered with 50 subjects. However, a post hoc analysis with the 7 variables in the model and 50 participants gave a high power of 91%, 96% and 100% for the three dependant variables SSS, Quick DASH and dexterity, respectively. Finally, the results suggest that the variables significantly explained variance in dexterity. But the results were not so satisfactory for the other 2 dependant variables, limiting the clinical application of the results.

Future recommendations would include performing a longitudinal study to understand the impact on change in function as this would help clinicians to determine the outcome of their treatments. Multiple sessions can be conducted instead of one long session to improve patient performance. Also, considering a population sample with varied severity of symptoms would help to understand how the predictability changes with changing severity of symptoms. Quick DASH was chosen as the self report functional outcome measure in this study as it has shown significant responsiveness in this population. But, other functional scales like Patient Rated Wrist and Hand Evaluation and Upper Extremity Functional Index can also be considered. This study thus lays the ground work for future research projects.

## CONCLUSION

This cross-sectional cohort study helps in predicting function for patients with Carpal Tunnel Syndrome considering dexterity as an important outcome. The results provide clinicians a set of 7 variables they can focus on while assessing patients with CTS waiting for surgery, instead of performing a wide battery of other tests. In addition to informing clinical practice, this study provides direction for future studies to streamline assessments and evaluation strategies for patients with CTS.

## Figures and Tables

**Table 1:** Patient characteristics.

Total number of participants	50
Gender distribution	23 males and 27 females
Age mean	58.2 ± 14.0 years
Hand dominance	Right hand- 46, Left hand- 4
Affected hand/hands	Bilateral- 36, Unilateral- 14
Time since diagnosis	4 months- 2 years

**Table 2:** Final regression model R^2^ and coefficients for dependant variable symptom severity scale score.

Model	R	R square	Adjusted R square	Standard error of the estimate
1	.647^a^	.418	.314	.58194
Model	Unstandardized coefficients B	Standardized coefficients Beta	t	Significance
(Constant)	3.433		6.435	.000
Age	-.012	-.240	-1.695	.098
Touch threshold of little finger, unaffected hand	.026	.251	1.884	.067
Vibration threshold of little finger, unaffected hand	-.006	-.098	-.652	.518
**Median/ ulnar Vibration ratio**	**.156**	**.279**	**2.130**	**.039**
Pain Tolerance Unaffected hand	-.012	-.150	-.722	.475
**Grip strength of affected hand**	**-.040**	**-.697**	**-2.045**	**.048**
Pinch strength of affected hand	.156	.558	1.852	.072

Predictors: (Constant), Pinch strength of affected hand, Touch threshold of little finger of the unaffected hand, Ratio of the median ulnar Vibration threshold of little finger in the unaffected hand, Age, PainTolerance of the Unaffected hand, Grip strength of affected hand. Only those were significant **bolded (p<0.05**); but all those that approach significance (p<0.10; highlighted) were brought forward into function modeling with the criteria that there have to be at least one variable representing each construct-hence one pain item also brought forward.

**Table 3:** Final regression Model R^2^ and coefficients for Quick DASH score.

Model	R	R square	Adjusted R square	Standard error of the estimate
1	.677^a^	.458	.361	16.13088
Model	Unstandardized coefficients B	Standardized coefficients Beta	t	Significance
(Constant)	51.601		3.489	.001
Age	-.104	-.072	-.526	.602
**Touch threshold of little finger, unaffected hand**	**1.232**	**.407**	**3.174**	**.003**
Vibration threshold of little finger, unaffected hand	-.244	-.148	-1.024	.312
Median/ ulnar Vibration ratio	3.452	.214	1.698	.097
Pain Tolerance Unaffected hand	.529	.233	1.160	.253
Grip strength of affected hand	-.574	-.345	-1.049	.301
Pinch strength of affected hand	-2.493	-.310	-1.065	.293

**Table 4:** coefficients for dexterity of affected hand.

Model	R	R square	Adjusted R square	Standard error of the estimate
1	.828^a^	.685	.629	14.58799
Model	Unstandardized coefficients B	Standardized coefficients Beta	t	Significance
(Constant)	18.335		1.371	0.18
**Age**	**.612**	**.356**	**3.427**	**0.001**
Touch threshold of little finger, unaffected hand	-.166	-.046	-.474	.648
**Vibration threshold of little finger, unaffected hand**	**1.166**	**.596**	**5.406**	**0.000**
Median/ ulnar Vibration ratio	-.650	-.034	-.354	0.73
Pain Tolerance Unaffected hand	-.107	-.040	-.261	0.80
Grip strength of affected hand	-.820	-.416	-1.658	0.10
Pinch strength of affected hand	1.675	.176	.792	0.43
